# Venous Thromboembolic Risk Does Not Increase After a Third Dose of SARS-CoV-2 mRNA-BNT162b2 Vaccine in Cancer Patients Receiving Active Systemic Therapies: Updated Results from the Vax-On-Third-Profile Study

**DOI:** 10.3390/vaccines13040392

**Published:** 2025-04-08

**Authors:** Fabrizio Nelli, Enzo Maria Ruggeri, Antonella Virtuoso, Diana Giannarelli, Jona Barbuta, Fabrizio Chegai, Armando Raso, Valentina Panichi, Julio Rodrigo Giron Berrios, Marta Schirripa, Cristina Fiore, Francesco Schietroma, Alessandro Strusi, Carlo Signorelli, Mario Giovanni Chilelli, Francesca Primi, Agnese Fabbri

**Affiliations:** 1 Department of Oncology and Hematology, Medical Oncology Unit, Central Hospital of Belcolle, 01100 Viterbo, Italyfrancesco.schietroma@asl.vt.it (F.S.);; 2 Biostatistics Unit, Scientific Directorate, Fondazione Policlinico Universitario A. Gemelli, IRCCS, 00136 Rome, Italy; 3 Department of Medicine, Vascular Diagnostics, Central Hospital of Belcolle, 01100 Viterbo, Italy; 4 Department of Oncology and Hematology, Thoracic and Interventional Radiology, Central Hospital of Belcolle, 01100 Viterbo, Italy; 5 Department of Oncology and Hematology, Citofluorimetry Unit, Central Hospital of Belcolle, 01100 Viterbo, Italy

**Keywords:** SARS-CoV-2, COVID-19, mRNA-BNT162b2 vaccine, third dose, solid tumors, active treatment, venous thromboembolism

## Abstract

(1) Background: Clinical evidence has raised concerns regarding a potential link between COVID-19 mRNA-based vaccines and the occurrence of thromboembolic events. So far, no research has explored the effects of this possible interaction in cancer patients undergoing active treatment. We leveraged prospective monitoring from the Vax-On-Third-Profile study to examine the development of venous thromboembolism (VTE) after the third dose of mRNA-BNT162b2 (tozinameran) and its association with antibody and lymphocyte responses. (2) Methods: Patients who had received a third dose of tozinameran and had not experienced any VTE in the previous 30 days were eligible. A serological evaluation was conducted before the booster vaccination (timepoint-1) and four weeks thereafter (timepoint-2) to measure antibody titers against the SARS-CoV-2 spike protein, as well as to determine the absolute counts of T-helper cells, T-cytotoxic cells, B cells, and NK cells. Data were acquired from November 2021 to October 2022 and analyzed from November 2022 to October 2023. (3) Results: The present study involved 429 patients who were given a third dose of tozinameran from 26 September to 30 October 2021. Among the active treatments of interest, 109 (25.4%) patients received targeted therapy, 111 (25.9%) received cytotoxic chemotherapy, 39 (9.1%) received immune checkpoint inhibitors, 21 (4.9%) received endocrine therapy, and 30 (7.0%) received a combination of chemotherapy and targeted agents in the eight weeks preceding the booster dosing. In addition, 119 (27.7%) patients who had discontinued any systemic therapy for at least 12 weeks accounted for the reference subgroup. After a median follow-up time of 10.6 (95% CI 8.1–11.7) months, we observed 31 venous thromboembolic events in the general population, for an overall incidence rate of 7.2% (95% CI 5.0–10.1). The median time to VTE development after booster immunization was 99 (95% CI 85–112) days. In a univariate comparison, patients exposed to targeted therapies (11.3% [95% CI 6.0–18.9]; *p* = 0.030) or immune checkpoint inhibitors (16.2% [95% CI 6.2–32.0]; *p* = 0.012) had a significantly higher incidence of VTE than the reference cohort (3.4% [95% CI 0.9–8.5]). Univariate analysis of immune responses showed that only dynamic changes pertaining to NK cell distributions correlated significantly with VTE occurrence. Multivariate regression analysis confirmed only a high-level NK cell response (OR 6.10 [9% CI 2.16–17.21]; *p* = 0.001), a history of thromboembolic events (OR 9.81 [3.99–24.13]; *p* < 0.001), and the presence of a central venous catheter (OR 5.02 [95% CI 1.84–13.67]; *p* = 0.002) as independently associated with an increased risk of VTE. (4) Conclusions: This prospective cohort study provides unprecedented evidence that cancer patients have no increased risk of developing VTE after the third dose of tozinameran, regardless of the type of active therapy. The specific pattern of lymphocyte response appears to increase thromboembolic risk, underlying immune dysregulation as a causal cofactor. These findings emphasize the need for additional monitoring after periodic COVID-19 vaccination in cancer patients.

## 1. Introduction

The coronavirus 2019 (COVID-19) pandemic has been a major health crisis, with over 770 million cases and around 7 million deaths worldwide by the end of 2023 [1]. Although the World Health Organization (WHO) has recently lowered the alert level [2], it still advises vaccination against SARS-CoV-2 infection as a priority for vulnerable individuals [3]. Among the latter, cancer patients receiving active treatments are a population of concern [4]. Even though booster doses of mRNA-based vaccines can shield these recipients from the severe effects of SARS-CoV-2 infection [5], waning immunity and the spread of new variants expose them to the continued risk of COVID-19 outbreaks [6]. As a result, expert groups from government authorities [7] and international societies recommend immunocompromised people with active cancer and survivors to receive the latest COVID-19 vaccines periodically [8,9].

A major complication of COVID-19 is the development of a systemic thrombo-inflammatory state [10]. Large population-based studies have confirmed the association between severe SARS-CoV-2 infection and increased risk of thromboembolic events [11]. Thrombosis associated with thrombocytopenia (or vaccine-induced prothrombotic immune thrombocytopenia) is a rare adverse event that has been reported in subjects who have received adenoviral-based vaccines encoding the SARS-CoV-2 spike protein [12]. This syndrome is associated with platelet-activating antibodies against platelet factor 4 [13]. Albeit to a lesser extent, thromboembolic events have also been observed following mRNA-based vaccinations [14]. Similar to the previous instance, epidemiologic studies have described an increased risk of pulmonary embolism with the mRNA-BNT162b2 (tozinameran) vaccine [15]. Unlike viral vector-based vaccines, the causal relationship between mRNA-based vaccination and thrombosis has not found viable explanations. Recent evidence suggests that changes in the vaccine-related immune response may underlie this association [16]. Based on these insights, mRNA-based immunization could induce a transient proinflammatory state resembling that of COVID-19 infection, thereby promoting the development of venous thromboembolism (VTE) under particular conditions [17,18]. Concerns that these events might be prevalent across all COVID-19 vaccine types have contributed to negative perceptions surrounding vaccine safety and increased hesitancy [19]. In this context, cancer patients undergoing active treatments represent a population of special interest for several reasons. Beyond being prime candidates to receive periodic boosters of the COVID-19 vaccine, their diagnosis of active cancer is an inherent predisposition for clot formation [20]. In addition, exposure to systemic cancer therapies is an established risk factor for VTE [21]. While the assumption of a causal link between mRNA-based COVID-19 vaccination and the development of VTE in actively treated cancer patients is conceivable, no studies have currently explored the clinical consequences of this putative interaction. We hypothesized that cancer patients exposed to high-risk systemic therapies experienced an increased incidence of VTE compared to cancer patients who discontinued any active treatment. Therefore, we leveraged the longitudinal monitoring of the Vax-On-Third-Profile study to examine the occurrence of VTE based on exposure to different systemic therapies. We also evaluated whether dynamic changes in antibody and peripheral lymphocyte responses following the third dose of tozinameran modulated the occurrence of VTE.

## 2. Materials and Methods

### 2.1. Study Design and Eligibility Criteria

We have previously reported the methodology and design of the Vax-On-Third-Profile study (EudraCT registration code: 2021-002611-54) [22]. The prospective design required close adherence to STROBE guidelines [23] and approval by the competent Ethics Committee (Institutional registration code: 1407/CE Lazio1). Prior to performing any procedure, all participants were asked to sign informed consent to process anonymized personal data for clinical research purposes. The current investigation involved a preplanned analysis of patients with histologically diagnosed solid tumors who received the third dose of tozinameran six months after vaccine priming through the initial two-dose schedule. Prospective assessment of VTE occurrence within 12 months after the booster dosing was the primary endpoint of this study. Adverse events of interest included deep vein thrombosis and pulmonary embolism. Data were acquired prospectively from November 2021 to October 2022 and analyzed from November 2022 to October 2023. During the observation period, no patients received additional vaccine boosters beyond the third dose of tozinameran. We compared the incidence of VTE in patients who had received different types of active treatments within eight weeks before booster vaccination to those who had discontinued any systemic therapy by at least 12 weeks (reference group). We assigned patients into five subgroups based on their active exposure to anticancer treatments: (1) targeted therapy (monoclonal antibodies and/or tyrosine kinase inhibitors), (2) cytotoxic chemotherapy, (3) immune checkpoint inhibitors, (4) endocrine therapy, or (5) a combination of chemotherapy and targeted agents. Patients in each subgroup were mutually exclusive. Confirmed diagnosis of previous SARS-CoV-2 infection at any time and VTE in the 30 days preceding the third dose of tozinameran were exclusion criteria for the current analysis. Participating patients were also analyzed for IgG antibody levels against the SARS-CoV-2 receptor-binding domain spike protein (RBD-S1) and counts of peripheral lymphocyte subsets. In this regard, the secondary endpoint was to assess whether changes in antibody titers and lymphocyte counts influenced the likelihood of developing VTE. Additional assessments included monitoring of SARS-CoV-2 infection at specific intervals or whenever it occurred after receipt of the third dose of tozinameran.

### 2.2. Diagnostic Assessments

Whole blood draws were collected immediately before the scheduled vaccination was given and again 28 days later. At both time points, the samples were processed promptly without storage to quantify anti-RBD-S1 IgG antibody serology and absolute counts of circulating lymphocyte subsets. In the first instance, we applied the SARS-CoV-2 IgG II Quant assay run on the ARCHITECT i2000sr automated platform (Abbott Core Diagnostics, Sligo, Ireland), as outlined by the producer [24]. According to WHO recommendations, we used binding antibody units (BAU)/mL as the standard measure of antibody serology [25]. In the second instance, the BD FACSCanto II system and BD FACSCanto clinical software (https://www.bdbiosciences.com/en-us/products/software/instrument-software/bd-facscanto-clinical-software (accessed on 15 February 2025), BD Biosciences, San Jose, CA, USA) enabled immunophenotyping of circulating lymphocytes. The complete antibody staining panel was supplied by BD Biosciences and included CD3 FITC, CD4 PE-Cy7, CD8 APC-Cy7, CD19 APC, CD45 PerCP-Cy5.5, CD16 PE, and CD56 PE. The BD Multitest 6-color TBNK reagent allowed quantification of absolute peripheral lymphocyte counts, including T helper cells (CD3^+^ and CD4^+^), T cytotoxic cells (CD3^+^ and CD8^+^), B cells (CD19^+^), and NK cells (CD16^+^ and CD56^+^). The procedure has already been described in detail and was conducted according to the producer’s instructions, as cited in [26]. We reported the corresponding values as absolute cell counts/µL for all lymphocyte subsets. During observation, the diagnosis of SARS-CoV-2 infection was defined by commercially available antigenic tests and/or polymerase chain reaction assays. We notified all confirmed cases of COVID-19 outbreaks to the agency for epidemiological surveillance [27].

### 2.3. Statistical Analysis

The current research applied SPSS version 23.0 (IBM SPSS Statistics for Windows, Armonk, NY, USA) for all statistical evaluations and Prism version 9.0 (GraphPad Software Inc., San Diego, CA, USA) for figure rendering. Descriptive statistics included a mean with standard deviation (SD) for continuous variables and frequencies (absolute and relative) with interquartile range (IQR) or 95% confidence interval (CI) for categorical variables. At both time points, we performed a multivariate analysis of antibody titers and lymphocyte subset counts through a generalized linear model based on their logarithmic (log) values. Independent covariates of this model included age, sex, Eastern Cooperative Oncology Group Performance Status (ECOG PS), cancer type, smoking habits, body mass index (BMI), disease staging, clinical setting of active treatment, corticosteroid intake, type of active treatment, adapted Khorana score, previous thromboembolic events, presence of vascular devices, antiplatelet, and anticoagulation therapies. To evaluate the sensitivity and specificity of antibody levels and absolute counts of lymphocyte subsets as predictors of VTE occurrence, we utilized a receiver operating characteristic (ROC) curve at the initial and at subsequent time points. We deemed immune markers to be reliable if they demonstrated a statistically significant correlation with the expected outcome and had an area under the curve (AUC) exceeding 0.80. Estimation of the Youden Index allowed the population to be dichotomized into two subgroups characterized by a low or high level of the relevant immune parameter [28]. Univariate comparative assessments were performed using Pearson’s *χ*^2^, the Mann–Whitney *U* test, or the Kruskal–Wallis test, as appropriate. Univariate comparisons between paired samples were performed by applying the Wilcoxon signed-rank test. A multivariate logistic regression analysis was conducted to determine the odds ratio (OR) for VTE occurrence, with a 95% confidence interval (CI). The multivariate model included clinical and immune covariates that showed a statistically significant association after univariate analysis. All statistics were two-tailed, and a *p* value less than 0.05 was deemed statistically significant.

## 3. Results

### 3.1. Patient Characteristics at Baseline

From 27 September to 30 October 2021, 429 patients received a third dose of tozinameran and were included in the analyses after confirming full compliance with the eligibility criteria. The median (SD) age was 67 (±11.2) years, and 58.7% were female. Breast (30.5%), colorectal (19.8%), and lung (19.1%) tumors were the most prevalent types of cancer. Current or former smoking habit was reported in 53.4%, and 22.2% of the recipients were found to be overweight (BMI ≥ 30). A total of 59.7% and 5.1% of the patients had metastatic disease and ECOG PS 2, respectively. Risk stratification according to the adapted Khorana score revealed 37.5% of participants at high risk of VTE. Additional risk factors included the presence of central venous devices in 24.2% and a history of thromboembolic events in 11% of the patients. Pre-vaccine antiplatelet or anticoagulation therapies were described in 26.1% and 18.6% of the patients, respectively. While heart disease was the most common therapeutic indication for both treatments, previous VTE and primary prophylaxis of cancer-related VTE accounted for 5.6% and 2.1% of the cases, respectively. Notably, no patients received additional antiplatelet or anticoagulant agents for the prevention of thromboembolic events potentially linked to vaccination. Among the active treatments of interest, 109 (25.4%) patients received targeted therapy, 111 (25.9%) received cytotoxic chemotherapy, 39 (9.1%) received immune checkpoint inhibitors, 21 (4.9%) received endocrine therapy, and 30 (7.0%) received a combination of chemotherapy and targeted agents in the eight weeks preceding the booster dosing. In addition, 119 (27.7%) patients who had discontinued any systemic therapy by at least 12 weeks served as the reference subgroup. Table 1 depicts the characteristics of the study populations at baseline.

### 3.2. Occurrence of Thromboembolic Events and SARS-CoV-2 Infections

After a median follow-up of 10.6 (95% CI 8.1–11.7) months, 31 patients experienced venous thromboembolic events, resulting in a cumulative incidence of 7.2% (95% CI 5.0–10.1). Confirmed VTE included deep vein thrombosis and pulmonary embolism in 21 (4.9%) and 10 (2.3%) cases, respectively. During the same time frame, 15 (3.5%) patients required at least one hospital admission, but we did not report any VTE-related deaths. The median time to development of VTE after the third dose of tozinameran was 99 (95% CI 85–112) days. Throughout the prospective observation period, we reported 126 cases of SARS-CoV-2 infection, resulting in an overall incidence rate of 29.4% (95% CI 26.2–35.3) and a median time to onset of 74 (95% CI 54–84) days. Only 10 (2.3% CI 1.4–4.4) patients experienced SARS-CoV-2 infection before the occurrence of VTE, with a median elapsing time of 60 (95% CI 32–84) days.

### 3.3. Analysis of Immune Correlates

**Figure 1:**
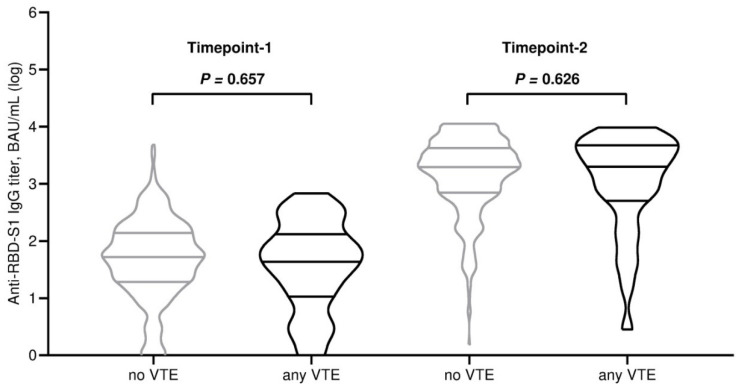
Comparison of plot distributions and medians of antibody titers. RBD-S1, receptor-binding domain (RBD) of the SARS-CoV-2 Spike protein (S1); BAU, binding antibody unit; log, logarithmic value; VTE, venous thromboembolism. Bars represent median values with interquartile range; timepoint-1 indicates the serological assessment preceding the third dose of tozinameran; timepoint-2 indicates the serological assessment performed four weeks following the third dose of tozinameran.

**Figure 2:**
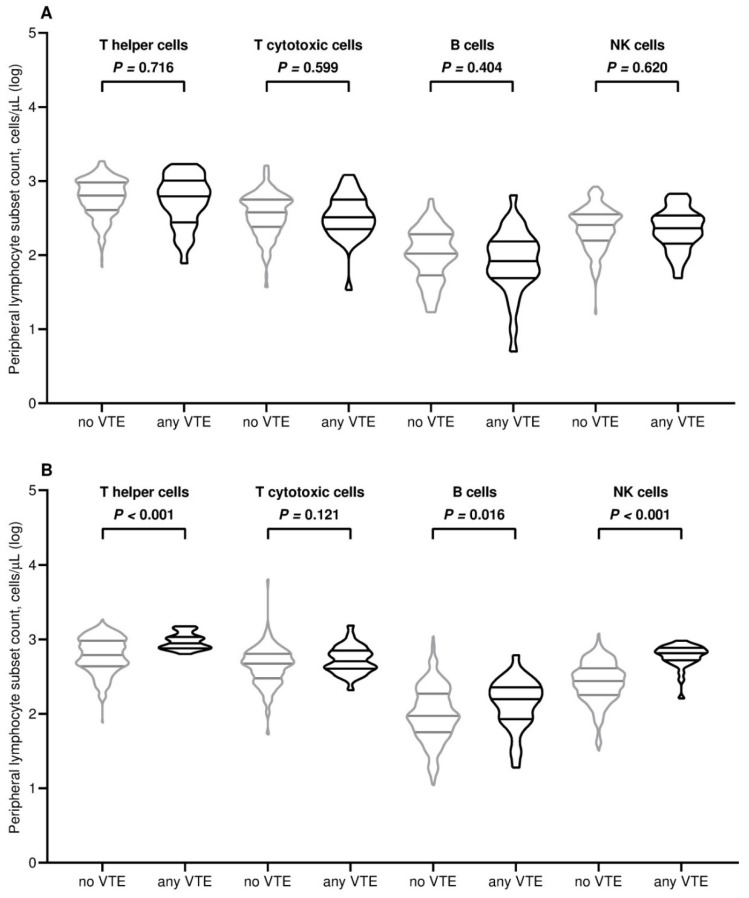
Comparison of the distribution of peripheral lymphocyte subpopulations at timepoint-1 (**A**) and timepoint-2 (**B**). Log, logarithmic value; VTE, venous thromboembolism. T helper cells, CD3^+^ and CD4^+^ cells; T cytotoxic cell, CD3^+^ and CD8^+^; B cells, CD19^+^; NK, natural killer CD16^+^ and CD56^+^; bars depict median values with interquartile range; timepoint-1 indicates the serological assessment preceding the third dose of tozinameran; timepoint-2 indicates the serological assessment performed four weeks following the third dose of tozinameran.

**Figure 3:**
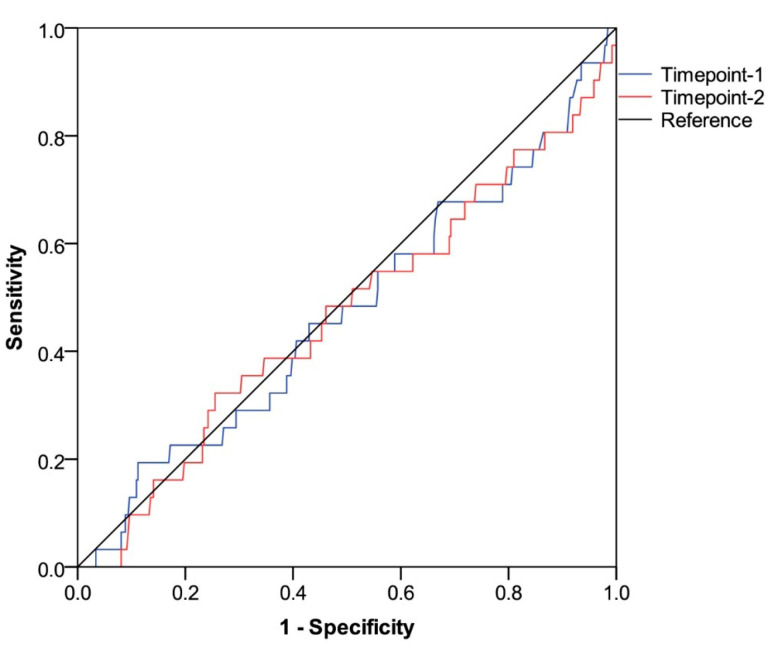
ROC curve analysis testing the potential of anti-RBD-S1 IgG (absolute titers) in predicting occurrence of VTE. AUC relative value at timepoint-1: 0.472 (95% CI 0.360–0.584), *p* = 0.609. AUC relative value at timepoint-2: 0.469 (95% CI 0.357–0.581), *p* = 0.566. Timepoint-1 indicates the serological assessment preceding the third dose of tozinameran; timepoint-2 indicates the serological assessment performed four weeks following the third dose of tozinameran. ROC, receiver operating characteristic; RBD-S1, receptor-binding domain (RBD) of the SARS-CoV-2 Spike protein (S1); AUC, area under the curve; CI, confidence interval; VTE, venous thromboembolism.

**Figure 4:**
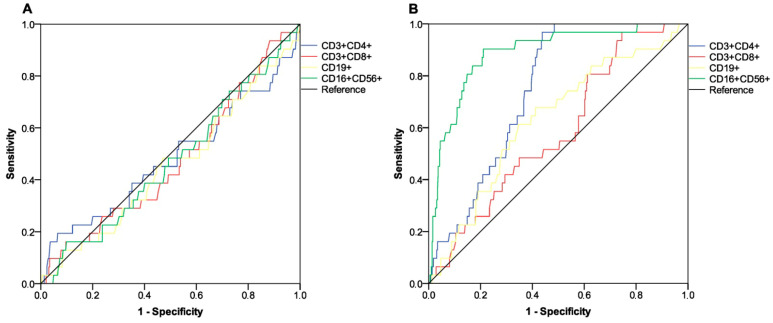
ROC curve analysis testing the potential of absolute counts of peripheral lymphocyte subpopulations in predicting the occurrence of VTE. (**A**) Timepoint-1; AUC relative values: T helper cells (CD3^+^ and CD4^+^): 0.482 (95% CI 0.361–0.604; *p* = 0.742); T cytotoxic cells (CD3^+^ and CD8^+^): 0.472 (95% CI 0.364–0.579; *p* = 0.601); B cells (CD19^+^): 0.457 (95% CI 0.348–0.566; *p* = 0.421); NK cells (CD16^+^ and CD56^+^): 0.473 (95% CI 0.366–0.579; *p* = 0.611). (**B**) Timepoint-2; AUC relative values: T helper cells (CD3^+^ and CD4^+^): 0.746 (95% CI 0.685–0.806; *p* < 0.001); T cytotoxic cells (CD3^+^ and CD8^+^): 0.584 (95% CI 0.489–0.678; *p* = 0.121; B cells (CD19^+^): 0.630 (95% CI 0.531–0.730; *p* = 0.016); NK cells (CD16^+^ and CD56^+^): 0.887 (95% CI 0.327–0.947; *p* < 0.001). Timepoint-1 indicates the serological assessment preceding the third dose of tozinameran; timepoint-2 indicates the serological assessment performed four weeks following the third dose of tozinameran. ROC, receiver operating characteristic; AUC, area under the curve; CI, confidence interval; VTE, venous thromboembolism.

All participants completed the assessment of antibody serology and lymphocyte subpopulation counts at baseline. By contrast, at the subsequent time point, 415 (96.7%) patients underwent full immunological evaluation due to early withdrawal in 14 (3.3%) cases for different reasons (Supplementary Figure S1). The third dose of tozinameran led to a significant rise in antibody levels within the general population and across all treatment subgroups (Supplementary Figures S2 and S3). The general distribution of peripheral lymphocyte subpopulations showed less predictable changes, with an increase in variability and median values that were statistically significant for T cytotoxic and NK cell subsets (Supplementary Figure S4). With the exclusion of patients receiving hormonal therapies, the significant increase in the latter subpopulations was confirmed by univariate assessment in all treatment subgroups (Supplementary Figure S5). At multivariate analysis, the generalized linear model confirmed that the different types of treatment had no impact on antibody titers after booster vaccination (Supplementary Table S1). The same model found only treatment with immune checkpoint inhibitors to be independently correlated with significantly increased counts of NK cells. (Supplementary Tables S2 and S3). Based on a univariate comparison at both time points, we found no difference in antibody titers between patients who experienced any VTE and those who did not (Table 2 and Figure 1). Similarly, the same comparative assessment at the first time point did not reveal any difference in the distribution of peripheral lymphocyte subpopulations (Table 2 and Figure 2A). By contrast, at the second time point, patients diagnosed with VTE showed significantly higher counts of T helper cells, B cells, and NK cells (Table 2 and Figure 2B). We then calculated a primary ROC curve to determine the potential of antibody titers in predicting the onset of VTE. The relevant AUC values at both time points were deemed unreliable (Figure 3). A subsequent ROC curve tested the correlation between absolute counts of circulating lymphocyte subsets and the same outcome. Only the AUC value related to the distribution of NK cells at time point 2 correlated reliably with the occurrence of VTE (Figure 4A,B). The Youden Index estimation pinpointed a value of 447/µL as the optimal threshold count, resulting in a sensitivity of 0.903 (95% CI 0.815–0.951) and specificity of 0.789 (95% CI 0.713–0.886). This cut point allowed us to divide patients into distinct subgroups of low (<447/µL) and high NK cell (≥447/µL) responders.

### 3.4. Assessment of Thromboembolic Risk

In the reference group, the incidence of VTE was 3.4% (95% CI 0.9–8.5). Based on a univariate comparison, patients receiving targeted therapies (11.3%; 95% CI 6.0–18.9) or immune checkpoint inhibitors (16.2%; 95% CI 6.2–32.0) had a significantly higher incidence of VTE than the reference subgroup. Conversely, the same figure did not differ in patients given hormonal therapies (5.0%; 95% CI 0.1–24.9), cytotoxic chemotherapy (6.7%; 95% CI 2.7–13.3), or chemotherapy combined with biological agents (3.5%; 95% CI 0.1–17.8). We also observed a significantly increased occurrence of VTE in high-level NK cell responders (25.7%; 95% CI 17.8–34.9) and patients experiencing early SARS-CoV-2 infection after booster vaccination. In addition to the latter covariates, univariate regression models confirmed exposure to immune checkpoint inhibitors and targeted therapies to be associated with a significantly higher risk of VTE. With the exclusion of the Khorana score, factors associated with a prothrombotic condition at baseline correlated uniformly with a significantly increased risk of VTE after the third dose of tozinameran. These included a history of thromboembolic events that had occurred at least 30 days earlier and the presence of a central venous catheter. It is noteworthy to observe that prior usage of antiplatelet or anticoagulant therapies did not reduce the cumulative incidence of VTE (Table 3). In the multivariate regression model for VTE, the relative risk did not differ significantly for patients with early SARS-CoV-2 infections and among those exposed to different active treatments compared to the reference subgroup. The same model confirmed the level of NK cell response and prothrombotic conditions correlated independently with a higher risk of VTE. Table 4 details the multivariate analysis of VTE risk assessment.

## 4. Discussion

The present research relied on a pre-specified analysis of the Vax-On-Third-Profile trial. The original study aimed to evaluate the immunogenicity and efficacy of the third dose of tozinameran depending on exposure to different types of active cancer treatments [22]. In this secondary analysis, we leveraged subsequent monitoring of participating recipients to observe the occurrence of VTE. Longitudinal observation during a median period of 10.6 months unveiled a cumulative incidence of 7.4% and an incidence rate of 8.2 cases per 100 person-years across the study population. We first reported these data after booster dosing of SARS-CoV-2 mRNA-based vaccines, and their mandatory interpretation remains challenging. The overall incidence of VTE did not differ from that found after vaccine priming through the initial two-dose schedule, but it was significantly higher than the same figure observed before the start of vaccination. This evidence and the median time to development of venous thromboembolic events (roughly three months) suggest a role for vaccination in the causal mechanism of VTE. A tentative interpretation could rely on a comparison with large-scale observational studies that assessed the risk of VTE after mRNA-based vaccination. These studies used the self-controlled case series (SCCS) design [29], which has been proposed as a standard approach to investigate the adverse events of vaccines [30]. This methodology cannot be applied to the current research because VTE in the previous six months could be attributable to the first two doses of vaccine, introducing an evaluation bias that cannot be overcome. In addition, most population-based studies performed the assessment after the first or second dose and did not include cancer patients [31]. However, a landmark study performed a subgroup analysis of generically definitive cancer patients. The authors described a cumulative incidence rate of venous thromboembolism of 1.3 per 100 person-years after the initial two-dose series of mRNA-based vaccines [32]. An indirect comparison suggests a more than sixfold increase in the risk of VTE in our case series. As relevant as this disproportion may seem, we must consider that Houghton et al. did not provide any information on cancer disease status or active treatments. These concerns would make a potential comparison with our data inconsistent. An additional key to interpretation could be based on a comparison with observational studies that have assessed the occurrence of thromboembolic events in patients with active cancer receiving systemic therapies. Although clearly indirect, such a comparative assessment seems more reliable in light of population characteristics similar to our case series. The most recent studies with an observation timeframe of at least 12 months have estimated a cumulative incidence of VTE between 6.6% and 7.7% [33,34], or from 5.8 to 9.6 per 100 person-years [35]. The latter figures appear consistent with our findings, suggesting that administration of the third dose of tozinameran did not affect the occurrence of VTE over a 12-month period.

This study aimed to evaluate the impact of different cancer treatments on the development of VTE after booster vaccination. Compared to those who had discontinued active treatment for at least three months, patients receiving targeted therapies or immune checkpoint inhibitors had more than threefold and nearly fivefold higher incidence of VTE, respectively. This was not unexpected, as these treatments have previously been linked to increased prothrombotic risk [36,37,38]. Although there was a possibility of speculating on a potential interaction between the third dose of vaccine and these treatments, the multivariate analysis did not confirm these findings. Apart from the established risk factors of central venous catheters [39] and prior thromboembolism [40], our multivariable testing only identified a rise in circulatory NK cells as a reliable predictor of VTE development. Recent studies have shown that NK cells play a key role in both the induction and effector phases of immune response following SARS-CoV-2 mRNA-based vaccination [41,42]. While their role in shaping immunity, and whether this effect is specific for mRNA vaccines, remains poorly understood [43], the data suggest that NK cell activation may drive some of the reactogenicity and contribute to vaccine-induced inflammatory symptoms [44]. Previous reports from the Vax-On-Third-Profile study have already shown considerable variability in immune response after the third dose of tozinameran in terms of changes in circulating lymphocyte subsets. These dynamic variations modulated clinical outcomes during active cancer treatment to different extents, including immune-related toxicities [45] and the onset of herpes zoster [46]. Since only immune checkpoint inhibitors had an impact on NK cell counts after booster vaccination, we can speculate that the third dose of tozinameran is the primary factor modulating dynamic changes in this subpopulation. According to our multivariate analysis, a high-level response in NK cell count would identify a subgroup of patients at increased thromboembolic risk. This finding is consistent with insights from a growing body of research suggesting that the production of venous thrombosis also involves the immune system through a complex interplay between innate and adaptive cells and endothelial cells [47]. In vivo experiments have found that upregulation of NK cells promotes neutrophil extracellular trap (NET)-dependent deep vein thrombosis through increased interferon-γ production. Conversely, it has been reported that specific depletion of NK cells protects against venous thrombosis through the inhibition of NET release [48]. Our results would also be consistent with a clinical study showing reduced expression of several inhibitory and activating receptors on NK cells in patients with pulmonary thromboembolism [49]. Although these findings appear suggestive, the clinical relevance is limited because of the paucity of evidence supporting their reliability. In addition, the observational nature of this study limits definitive conclusions about whether changes in NK cell responses following vaccination directly contribute to VTE. The uncertainty of this potential interaction would benefit from further validation through larger, controlled studies or mechanistic investigations to elucidate the biological pathways linking COVID-19 vaccination, immune modulation, and venous thromboembolic risk.

Of course, this study acknowledges several limitations, which may extend beyond the following issues. First, patient recruitment was not stratified according to the type of active treatment. Instead, we applied “all-comer” enrollment with the aim of providing vaccination to as many eligible patients as quickly as possible during the COVID-19-related emergency. This model implied wide variability in the size of the various subgroups of different systemic therapies. Such a disproportion may have had a nonnegligible impact on the validity of the univariate analysis. Second, the current research design did not allow us to apply the SCCS methodology, preventing direct comparative assessments. Comparisons with historical data prior to the start of vaccination may be unreliable for the purposes of this study, as some patients had not yet received a cancer diagnosis and most were not on active treatment. Third, the research project of the Vax-On-Third-Profile study relied on the immunophenotyping of peripheral lymphocytes as a correlate of changes in cellular immunity induced by the booster dose of tozinameran. Although highly standardized and easily accessible, the viability of this analysis methodology in the context of SARS-CoV-2 vaccine immunogenicity has been only suggested and remains undefined [50,51]. Finally, the heterogeneity of our case series warranted a comprehensive analysis including all factors with potential prognostic value. This may have mitigated the risk of false-negative results in an unprecedented experimental setting. However, we cannot overlook the inflation of alpha risk due to multiple univariate comparisons and the likelihood of false-positive results that is inherent to this methodology.

## 5. Conclusions

The present study is the first prospective study to address the incidence of VTE after SARS-CoV-2 mRNA-based vaccination in actively treated cancer patients. Our comprehensive analyses and comparisons with available evidence endorse the hypothesis that booster dosing does not influence the risk of VTE. While the causal relationship between SARS-CoV-2 mRNA-based vaccines and thromboembolism remains uncertain, periodic booster vaccinations are the most effective preventive measure against severe sequelae of COVID-19 in immunocompromised individuals. From a clinical standpoint, treating physicians should provide additional monitoring for cancer patients at higher thrombotic risk, including those with central venous devices and a history of thromboembolic events, regardless of their systemic therapy.

## Figures and Tables

**Table 1:** Patient characteristics at baseline.

Characteristic	All Patients, N = 429 (100%)
Median age, years (SD)	67.0 (±11.2)
- <65 years	200 (46.6%)
- ≥65 years	229 (53.4%)
Sex	
- female	252 (58.7%)
- male	177 (41.3%)
Primary tumor diagnosis	
- breast	131 (30.5%)
- lung	82 (19.1%)
- kidney	13 (3.0%)
- prostate	11 (2.6%)
- colorectal	85 (19.8%)
- urothelial	17 (4.0%)
- pancreatic	14 (3.3%)
- gastric	17 (4.0%)
- skin (Melanoma, Merkel-cell)	10 (2.3%)
- gynecological	15 (3.5%)
- head and neck	5 (1.2%)
- brain	15 (3.5%)
- others ^a^	14 (3.3%)
Smoking habit	
- never	200 (46.6%)
- current or former	229 (53.4%)
BMI	
- median (SD)	25.3 (4.8)
- ≥30 kg/m^2^	95 (22.2%)
Disease staging	
- localized or locally advanced	173 (40.3%)
- metastatic	256 (59.7%)
Treatment setting	
- (neo)adjuvant	176 (41.0%)
- metastatic (first or later line)	253 (59.0%)
ECOG PS	
- 0	220 (51.3%)
- 1	187 (46.3%)
- 2	22 (5.1%)
Corticosteroid therapy ^b^	62 (14.5%)
Active systemic therapy	
- none (reference subgroup)	119 (27.7%)
- targeted therapy	109 (25.4%)
- cytotoxic chemotherapy	111 (25.9%)
- immune checkpoint inhibitors	39 (9.1%)
- hormonal therapy	21 (4.9%)
- cytotoxic chemotherapy and targeted agents	30 (7.0%)
Khorana score ^c^	
- high risk	161 (37.5%)
- low risk	268 (62.5%)
Presence of CVC	104 (24.2%)
Previous VTE	
- any	47 (11.0%)
- after the first or second of vaccine	30 (7.0%)
- before the start of vaccination	17 (3.9%)
- none	382 (89.0%)
Antiplatelet therapies ^d^	112 (26.1%)
Therapeutic indication for antiplatelet agents	
- heart disease	57 (13.3%)
- cerebrovascular disease	23 (5.4%)
- peripheral artery disease	15 (3.5%)
- previous VTE	12 (2.8%)
- hematologic disease	3 (0.7%)
- others	2 (0.5%)
Anticoagulation therapies ^e^	80 (18.7%)
Therapeutic indication for anticoagulant agents	
- heart disease	49 (11.4%)
- previous VTE	12 (2.8%)
- primary prophylaxis of cancer-related VTE	9 (2.1%)
- cerebrovascular disease	7 (1.6%)
- others	3 (0.7%)

^a^ Other cancer diagnoses included soft-tissue sarcoma, thymoma, testicular cancer, hepatocellular carcinoma, biliary tract cancer, esophageal cancer, and GIST; ^b^ corticosteroid therapy denotes ≥10 mg of prednisone equivalent daily lasting ≥7 days before the third dose of tozinameran (excluding premedication for chemotherapy); ^c^ adapted from Khorana score (cancer-related high risk for venous thromboembolism included active diagnosis of gastric, esophagus, lung, pancreatic, gynecological, germ-cell, kidney, and bladder tumors; low risk types for venous thromboembolism include other conditions of solid neoplasm diagnosis); ^d^ antiplatelet therapies included use of any dose of aspirin, ticlopidine, clopidogrel, or ticagrelor; ^e^ anticoagulation therapies included use of any dose of low-molecular-weight heparin, apixaban, edoxaban, rivaroxaban, or dabigatran etexilate. SD, standard deviation; ECOG PS, Eastern Cooperative Oncology Group Performance Status; BMI, body mass index; VTE, venous thromboembolism; CVC, central venous catheter.

**Table 2:** Correlates of immune response.

Parameter	Timepoint-1				Timepoint-2			
	All Patients (N = 429)	No VTE (N = 384)	Any VTE (N = 31)	*p* Value	All Patients (N = 415)	No VTE (N = 289)	Any VTE (N = 31)	*p* Value
Anti-RDB-S1 antibody titer (BAU/mL), median (95% CI)	53 (44–61)	53 (45–62)	43 (27–80)	0.609	2064 (1768–2257)	2066 (1755–2282)	1981 (809–3570)	0.566
T helper cell count/µL, median (95% CI)	639 (601–680)	639 (591–688)	623 (439–792)	0.742	680 (610–737)	615 (583–689)	882 (786–1040)	<0.001
T cytotoxic cell count/µL, median (95% CI)	376 (343–392)	345 (338–393)	325 (252–394)	0.601	471 (452–507)	471 (452–507)	509 (419–639)	0.121
B cell/µL, median (95% CI)	100 (95–106)	100 (96–106)	83 (57–118)	0.421	97 (92–107)	94 (90–103)	157 (111–222)	0.016
NK cell count/µL, median (95% CI)	256 (230–274)	256 (230–274)	231 (181–313)	0.611	292 (266–311)	275 (252–299)	653 (561–692)	<0.001

T helper cells, CD3^+^ and CD4^+^ cells; T cytotoxic cell, CD3^+^ and CD8^+^; B cells, CD19^+^; NK, Natural killer, CD16^+^ and CD56^+^; timepoint-1 indicates the serological assessment preceding the third dose of tozinameran; timepoint-2 indicates the serological assessment performed four weeks following the third dose of tozinameran. VTE, venous thromboembolism; RBD-S1, receptor-binding domain (RBD) of the SARS-CoV-2 Spike protein (S1); BAU, binding antibody unit; CI, confidence interval.

**Table 3:** Univariate analysis of venous thromboembolism.

Covariates	No TEEs (N = 384)	Any TEEs (N = 31)	OR (95% CI)	*p* Value
Age				
- <65 years (N = 198)	179 (90.4%)	19 (9.6%)	1.00	-
- ≥65 years (N = 217)	205 (94.5%)	12 (5.5%)	0.53 (0.25–1.13)	0.102
Sex				
- female (N = 244)	229 (93.9%)	15 (6.1%)	1.00	-
- male (N = 171)	155 (90.6%)	16 (9.4%)	1.57 (0.75–3.21)	0.224
Tumor type				
- breast cancer (N = 128)	118 (92.2%)	10 (7.8%)	1.00	-
- lung cancer (N = 79)	72 (91.1%)	7 (8.9%)	1.14 (0.41–3.14)	0.790
- colorectal cancer (N = 41)	37 (90.2%)	4 (9.8%)	1.27 (0.37–4.30)	0.695
- genitourinary cancer (N = 86)	80 (93.0%)	6 (7.0%)	0.88 (0.30–2.53)	0.820
- others (N = 81)	77 (95.1%)	4 (4.9%)	0.61 (0.18–2.02)	0.422
ECOG PS				
- 0 (N = 215)	198 (91.2%)	17 (7.9%)	1.00	-
- 1 (N = 179)	165 (92.2%)	14 (7.8%)	0.98 (0.47–2.06)	0.975
- 2 (N = 21)	21 (100%)	-	1.00 (NA)	0.998
Smoking habits				
- never (N = 193)	178 (92.2%)	15 (7.8%)	1.00	-
- former or current (N = 222)	206 (92.8%)	16 (7.2%)	0.92 (.044–1.91)	0.827
BMI				
- <30 (N = 326)	303 (93.0%)	23 (7.0%)	1.00	-
- ≥30 (N = 89)	81 (91.0%)	8 (9.0%)	1.31 (0.56–3.05)	0.521
Disease staging				
- early stage (N = 166)	156 (94.0%)	10 (6.0%)	1.00	-
- advanced stage (N = 249)	228 (91.6%)	21 (8.4%)	1.43 (0.65–3.13)	0.362
Therapeutic setting				
- adjuvant or neoadjuvant (N = 169)	158 (93.5%)	11 (6.5%)	1.00	-
- metastatic, any line of therapy (N = 246)	226 (91.9%)	20 (8.1%)	1.27 (0.59–2.72)	0.538
Type of active treatment				
- none (N = 118)	114 (96.6%)	4 (3.4%)	1.00	-
- targeted therapies (N = 106)	94 (88.7%)	12 (11.3%)	3.63 (1.13–11.65)	0.030
- cytotoxic chemotherapy (N = 105)	98 (93.3%)	7 (6.7%)	2.03 (0.57–7.16)	0.268
- immune checkpoint inhibitors (N = 37)	31 (83.8%)	6 (16.2%)	5.51 (1.46–20.77)	0.012
- hormonal therapies (N = 20)	19 (95.0%)	1 (5.0%)	1.50 (0.15–14.15)	0.723
- chemotherapy and targeted agents (N = 29)	28 (96.5%)	1 (3.5%)	1.01 (0.10–9.46)	0.988
Corticosteroid therapy ^a^				
- none (N = 354)	329 (92.9%)	25 (7.1%)	1.00	-
- any (N = 61)	55 (90.2%)	6 (9.8%)	1.43 (0.56–3.65)	0.449
Khorana score ^b^				
- low risk (N = 260)	238 (91.5%)	22 (8.5%)	1.00	-
- high risk (N = 155)	146 (94.2%)	9 (5.8%)	0.66 (0.29–1.48)	0.322
Previous VTE				
- none (N = 369)	361 (97.8%)	8 (2.2%)	1.00	-
- any (N = 46)	23 (50.0%)	23 (50.0%)	14.78 (7.77–28.10)	<0.001
CVC				
- no (N = 316)	303 (95.9%)	13 (4.1%)	1.00	-
- yes (N = 99)	81 (81.8%)	18 (18.2%)	5.17 (2.43–11.01)	<0.001
Antiplatelet agents ^c^				
- no (N = 306)	283 (92.5%)	23 (7.5%)	1.00	-
- yes (N = 109)	101 (92.7%)	8 (7.3%)	0.97 (0.42–2.24)	0.952
Anticoagulation therapy ^d^				
- no (N = 336)	313 (93.2%)	23 (6.8%)	1.00	-
- yes (N = 78)	70 (89.7%)	8 (10.3%)	1.55 (0.66–3.62)	0.306
Previous SARS-CoV-2 infection				
- no (N = 405)	383 (94.6%)	22 (5.4%)	1.00	-
- yes (N = 10)	1 (10.0%)	9 (90.0%)	35.65 (8.1–>100)	<0.001
NK cell count ^e^				
- low responders (N = 306)	303 (99.0%)	3 (1.0%)	1.00	-
- high responders (N = 108)	81 (74.1%)	28 (25.9%)	12.33 (5.22–29.13)	<0.001

^a^ Corticosteroid therapy denotes ≥10 mg of prednisone equivalent daily lasting ≥7 days before the third dose of tozinameran (excluding premedication for chemotherapy); ^b^ adapted from Khorana score (cancer-related high risk for venous thromboembolism included active diagnosis of gastric, esophagus, lung, pancreatic, gynecological, germ-cell, kidney, and bladder tumors; low risk types for venous thromboembolism included other conditions of solid neoplasm diagnosis); ^c^ antiplatelet therapies included use of any dose of aspirin, ticlopidine, clopidogrel, or ticagrelor; ^d^ anticoagulation therapies included use of any dose of low-molecular-weight heparin, apixaban, edoxaban, rivaroxaban, or dabigatran etexilate; ^e^ low-responders accounted for the subgroup of patients with NK cell count <447/µL, high-responders accounted for the subgroup of patients with NK cell count ≥447/µL. ECOG PS, Eastern Cooperative Oncology Group Performance Status; BMI, body mass index; VTE, venous thromboembolism; CVC, central venous catheter; OR, odds ratio; CI, confidence interval.

**Table 4:** Multivariate analysis of venous thromboembolism.

Covariates	OR (95% CI)	*p* Value
Type of active treatment		
- none (reference)	1.00	-
- targeted therapies	1.23 (0.18–8.45)	0.830
- cytotoxic chemotherapy	0.73 (0.11–4.73)	0.748
- immune checkpoint inhibitors	0.63 (0.10–3.92)	0.624
- hormonal therapies	0.79 (0.13–4.71)	0.796
- chemotherapy and targeted agents	1.01 (0.23–4.32)	0.990
Previous VTE		
- none (reference)	1.00	-
- any	9.81 (3.99–24.13)	<0.001
CVC		
- no (reference)	1.00	-
- yes	5.02 (1.84–13.67)	0.002
Previous SARS-CoV-2 infection		
- no (reference)	1.00	-
- yes	1.19 (0.40–3.09)	0.828
NK cell count ^a^		
- low responders (reference)	1.00	-
- high responders	6.10 (2.16–17.21)	0.001

^a^ low-responders accounted for the subgroup of patients with NK cell count <447/µL, high-responders accounted for the subgroup of patients with NK cell count ≥447/µL. VTE, venous thromboembolism; CVC, central venous catheter; OR, odds ratio; CI, confidence interval.

## Data Availability

The data that support the findings of this study are available from the corresponding author upon reasonable request.

## Supplementary Materials

The following supporting information can be downloaded at: https://www.mdpi.com/article/10.3390/vaccines13040392/s1.
