# Supplementary material for: Venous Thromboembolic Risk Does Not Increase After a Third Dose of SARS-CoV-2 mRNA-BNT162b2 Vaccine in Cancer Patients Receiving Active Systemic Therapies: Updated Results from the Vax-On-Third-Profile Study
Source: Vaccines (Basel). 2025 Apr 8;13(4):392. doi: 10.3390/vaccines13040392 (PMC12031221; doi:10.3390/vaccines13040392)
Supplement: Supplementary file 1 [file vaccines-13-00392-s001.zip › vaccines-3507596-supplementary.pdf]

SUPPLEMENTARY MATERIAL

Title

Venous thromboembolic risk does not increase after a third dose of SARS-CoV-2 mRNA-BNT162b2 vaccine in cancer patients receiving active systemic therapies: updated results from the Vax-On-Third-Profile study.

Fabrizio Nelli [1], Enzo Maria Ruggeri [1], Antonella Virtuoso [1], Diana Giannarelli [2], Jona Barbuta [3], Fabrizio Chegai [4], Armando Raso [4], Valentina Panichi [5], Julio Rodrigo Giron Berrios [1], Marta Schirripa [1], Cristina Fiore [1], Francesco Schietroma [1], Alessandro Strusi [1], Carlo Signorelli [1], Mario Giovanni Chilelli [1], Francesca Primi [1], and Agnese Fabbri [1]

[1] Department of Oncology and Hematology, Medical Oncology Unit, Central Hospital of Belcolle, Viterbo, Italy

[2] Biostatistics Unit, Scientific Directorate, Fondazione Policlinico Universitario A. Gemelli, IRCCS, Rome, Italy

[3] Department of Medicine, Vascular Diagnostics, Central Hospital of Belcolle, Viterbo, Italy

[4] Department of Oncology and Hematology, Thoracic and Interventional Radiology, Central Hospital of Belcolle, Viterbo, Italy

[5] Department of Oncology and Hematology, Citofluorimetry Unit, Central Hospital of Belcolle, Viterbo, Italy

*** Correspondence:** Fabrizio Nelli, MD; Department of Oncology and Hematology, Medical Oncology Unit, Central Hospital of Belcolle, Strada Sammartinese snc, 01100 Viterbo, Italy, Phone +390761339055, Fax +390761339039, e-mail: fabrizio.nelli@asl.vt.it, ORCID iD: 0000-0001-8374-1362

Supplementary Table S1. Multivariate analysis of antibody response

Covariate	Anti-RBD-S1 antibody titer at timepoint-1 (log)		Anti-RBD-S1 antibody titer at timepoint-2 (log)	
	Beta (95% CI)	P value	Beta (95% CI)	P value
Sex				
- male vs. female	-0.13 (-0.30 to 0.03)	0.108	-0.01 (-0.17 to 0.14)	0.825
Age (years)				
- >65 vs. ≤65	-0.07 (-0.20 to 0.06)	0.296	0.03 (-0.09 to 0.16)	0.614
Tumor type				
- breast cancer	-	-	-	-
- lung cancer	-0.54 (-0.90 to -0.19)	0.002	-0.52 (-0.87 to -0.18)	0.003
- colorectal cancer	-0.41 (-0.71 to -0.10)	0.009	-0.43 (-0.73 to -0.13)	0.004
- genitourinary cancer	-0.52 (-0.79 to -0.25)	<0.001	-0.30 (-0.56 to -0.04)	0.023
- others	-0.21 (-0.42 to 0.01)	0.055	-0.14 (-0.34 to 0.06)	0.181
ECOG PS				
- 0	-	-	-	-
- 1	-0.06 (-0.21 to 0.07)	0.344	-0.01 (-0.15 to 0.12)	0.857
- 2	-0.45 (-0.76 to -0.13)	0.005	-0.43 (-0.74 to -0.13)	0.005
Smoking habits				
- ever vs. never	-0.05 (-0.20 to 0.08)	0.437	0.07 (-0.06 to 0.21)	0.313
BMI				
- ≥30 vs. <30	-0.08 (-0.024 vs. 0.06)	0.272	-0.04 (-0.20 to 0.10)	0.523
Disease staging				
- advanced vs. early stage	0.06 (-0.19 to 0.32)	0.631	0.07 (-0.24 to 0.26)	0.959
Treatment setting				
- metastatic vs. (neo)adjuvant	-0.02 (-0.28 to 0.23)	0.874	-0.04 (-0.29 to 0.21)	0.740
Type of active treatment				
- none (reference subgroup)	-	-	-	-
- targeted therapy	0.08 (-0.12 to 0.30)	0.434	0.02 (-0.18 to 0.23)	0.798
- cytotoxic chemotherapy	0.12 (-0.07 to 0.31)	0.211	0.05 (-0.13 to 0.24)	0.552
- immune checkpoint inhibitors	-0.07 (-0.36 to 0.22)	0.628	-0.01 (-0.29 to 0.27)	0.948
- hormonal therapy	0.01 (-0.31 to 0.35)	0.912	0.17 (-0.15 to 0.49)	0.302
- cytotoxic chemotherapy and targeted agents	-0.13 (-0.43 to 0.17)	0.401	-0.23 (-0.52 to 0.06)	0.126
Corticosteroid therapy [a]				
- yes vs. none	-0.40 (-0.59 to -0.21)	<0.001	-0.13 (-0.31 to 0.04)	0.152

Khorana score [b]<br>- high vs. low risk	0.37 (0.12 to 0.63)	0.004	0.04 (-0.19 to 0.29)	0.699
Previous TEEs<br>- yes vs. none	-0.10 (-0.35 to 0.14)	0.407	-0.17 (-0.42 to 0.06)	0.155
CVC<br>- yes vs. no	0.10 (-0.05 to 0.26)	0.205	0.09 (-0.06 to 0.24)	0.254
Antiplatelet agents [c]<br>- yes vs. no	-0.04 (-0.15 to 0.14)	0.958	0.01 (-0.14 to 0.14)	0.968
Anticoagulation therapy [d]<br>- yes vs. no	-0.04 (0.20 to 0.12)	0.634	-0.01 (-0.16 to 0.15)	0.964

RBD-S1, receptor-binding domain (RBD) of the SARS-CoV-2 Spike protein (S1); log, logarithmic values; CI, confidence intervals; ECOG PS, Eastern Cooperative Oncology Group Performance Status; BMI, body mass index; TEEs, thromboembolic events; CVC, central venous catheter. [a] Corticosteroid therapy indicates ≥10 mg daily of prednisone or equivalent for at least 7 days before the third dose of tozinameran (excluding premedication for chemotherapy); [b] adapted from Khorana score (cancer-related high risk for venous thromboembolism included active diagnosis of gastric, esophagus, lung, pancreatic, gynecological, germ-cell, kidney, and bladder tumors; low risk types for venous thromboembolism include other conditions of solid neoplasm diagnosis); [c] antiplatelet therapies included the use of any dose aspirin, ticlopidine, clopidogrel, or ticagrelor; [d] anticoagulation therapies included the use of any dose low-molecular-weight heparin, apixaban, edoxaban, rivaroxaban, or dabigatran etexilate; timepoint-1 indicates assessment before the third dose of tozinameran; timepoint-2 indicates assessment four weeks after the third dose of tozinameran. *P* values relied on parametric two-sided Wald's $\chi 2$ test with Bonferroni (α =0.01) correction for multiple comparisons. A two-sided *P* value of <0.05 was considered statistically significant.

Supplementary Table S2. Multivariate analysis of peripheral lymphocyte counts at timepoint-1

Covariate	T helper cell count (log)		T cytotoxic cell count (log)		B cell count (log)		NK cell count (log)	
	Beta (95% CI)	P value	Beta (95% CI)	P value	Beta (95% CI)	P value	Beta (95% CI)	P value
Sex<br>- male vs. female	-0.03 (-0.09 to 0.02)	0.257	-0.05 (-0.11 to 0.01)	0.137	-0.07 (-0.15 to 0.01)	0.107	0.05 (-0.01 to 0.11)	0.139
Age (years)<br>- >65 vs. ≤65	0.19 (-0.02 to 0.06)	0.437	0.01 (-0.04 to 0.06)	0.608	0.01 (-0.05 to 0.08)	0695	0.08 (0.02 to 0.13)	0.004
Tumor type								
- breast cancer	-	-	-	-	-	-	-	-
- lung cancer	-0.05 (-0.18 to 0.07)	0.385	-0.02 (-0.17 to -0.11)	0.712	-0.11 (-0.29 to 0.07)	0.241	0.01 (-0.12 to 0.16)	0.821
- colorectal cancer	-0.04 (-0.15 to 0.06)	0.452	0.04 (-0.08 to 0.16)	0.508	0.03 (-0.12 to 0.19)	0.667	0.03 (-0.09 to 0.16)	0.574
- genitourinary cancer	0.01 (-0.07 to 0.16)	0.697	0.06 (-0.04 to -0.17)	0.267	0.05 (-0.08 to 0.19)	0.440	0.08 (-0.03 to 0.19)	0.155
- others	0.04 (-0.03 to 0.12)	0.229	0.04 (-0.04 to 0.13)	0.300	0.15 (0.03 to 0.26)	0.008	0.05 (-0.03 to 0.13)	0.266
ECOG PS								
- 0	-	-	-	-	-	-	-	-
- 1	-0.01 (-0.06 to 0.04)	0.656	-0.08 (-0.13 to -0.02)	0.007	-0.01 (-0.08 to 0.06)	0.863	0.02 (-0.03 to 0.08)	0.341
- 2	-0.03 (-0.14 to 0.08)	0.594	-0.02 (-0.14 to -0.10)	0.762	0.03 (-0.12 to 0.19)	0.658	-0.06 (-0.18 to 0.06)	0.357
Smoking habits<br>- ever vs. never	0.01 (-0.04 to 0.05)	0.882	0.02 (-0.03 to 0.08)	0.458	0.06 (-0.01 to 0.14)	0.079	0.02 (-0.03 to 0.08)	0.387
BMI<br>- ≥30 vs. <30	0.03 (-0.01 vs. 0.09)	0.193	0.05 (-0.01 to 0.11)	0.115	0.01 (-0.08 to 0.08)	0.995	0.02 (-0.04 to 0.08)	0.515
Disease staging<br>- advanced vs. early stage	0.01 (-0.09 to 0.10)	0.915	-0.01 (-0.11 to 0.10)	0.946	0.01 (-0.12 to 0.14)	0.902	-0.01 (-0.11 to 0.06)	0.875
Treatment setting<br>- metastatic vs. (neo)adjuvant	-0.07 (-0.17 to 0.01)	0.119	-0.07 (-0.17 to 0.03)	0.181	-0.04 (-0.18 to 0.09)	0.528	-0.02 (-0.13 to 0.08)	0.613
Type of active treatment								
- none (reference subgroup)	-	-	-	-	-	-	-	-
- targeted therapy	0.09 (-0.01 to 0.17)	0.055	0.08 (-0.01 to 0.17)	0.054	0.11 (-0.01 to 0.22)	0.058	0.04 (-0.04 to 0.13)	0.325
- cytotoxic chemotherapy	0.01 (-0.07 to 0.06)	0.996	-0.04 (-0.11 to 0.03)	0.315	0.01 (-0.09 to 0.10)	0.883	-0.10 (-0.18 to 0.02)	0.057
- immune checkpoint inhibitors	-0.02 (-0.13 to 0.07)	0.595	0.03 (-0.08 to 0.15)	0.573	0.03 (-0.11 to 0.18)	0.642	-0.07 (-0.19 to 0.04)	0.217
- hormonal therapy	0.01 (-0.11 to 0.13)	0.855	0.08 (-0.14 to 0.31)	0.060	0.18 (0.01 to 0.36)	0.038	0.07 (-0.06 to 0.21)	0.299
- cytotoxic chemotherapy and targeted agents	0.03 (-0.07 to 0.14)	0.557	0.02 (-0.09 to 0.15)	0.650	0.01 (-0.14 to 0.17)	0.828	-0.06 (-0.18 to 0.06)	0.321
Corticosteroid therapy [a]								

- yes vs. none	-0.20 (-0.27 to -0.14)	<0.001	-0.02 (-0.10 to 0.04)	0.453	-0.09 (-0.19 to 0.01)	0.055	-0.03 (-0.11 to 0.03)	0.331
Khorana score [b]<br>- high vs. low risk	0.06 (-0.02 to 0.15)	0.170	0.03 (-0.07 to 0.13)	0.535	0.01 (-0.12 to 0.14)	0.880	-0.08 (-0.18 to 0.01)	0.109
Previous TEEs<br>- yes vs. none	0.04 (-0.03 to 0.11)	0.294	0.03 (-0.04 to 0.12)	0.390	0.03 (-0.07 to 0.14)	0.499	-0.03 (-0.12 to 0.05)	0.418
CVC<br>- yes vs. no	0.04 (-0.01 to 0.10)	0.096	0.04 (-0.01 to 0.11)	0.159	-0.01 (-0.09 to 0.06)	0.732	0.01 (-0.04 to 0.08)	0.577
Antiplatelet agents [c]<br>- yes vs. no	-0.01 (-0.07 to 0.03)	0.562	-0.02 (-0.08 to 0.03)	0.415	-0.03 (-0.11 to 0.04)	0.428	0.01 (-0.04 to 0.07)	0.598
Anticoagulation therapy [d]<br>- yes vs. no	-0.02 (-0.08 to 0.03)	0.364	-0.02 (-0.09 to 0.04)	0.546	0.01 (-0.07 to 0.09)	0.793	0.01 (-0.05 to 0.07)	0.777

Log, logarithmic values; CI, confidence intervals; ECOG PS, Eastern Cooperative Oncology Group Performance Status; BMI, body mass index; TEEs, thromboembolic events; CVC, central venous catheter. [a] Corticosteroid therapy indicates ≥10 mg daily of prednisone or equivalent for at least 7 days before the third dose of tozinameran (excluding premedication for chemotherapy); [b] adapted from Khorana score (cancer-related high risk for venous thromboembolism included active diagnosis of gastric, esophagus, lung, pancreatic, gynecological, germ-cell, kidney, and bladder tumors; low risk types for venous thromboembolism include other conditions of solid neoplasm diagnosis); [c] antiplatelet therapies included the use of any dose aspirin, ticlopidine, clopidogrel, or ticagrelor; [d] anticoagulation therapies included the use of any dose low-molecular-weight heparin, apixaban, edoxaban, rivaroxaban, or dabigatran etexilate; T helper cells, $CD3^+CD4^+$ cells; T cytotoxic cell, $CD3^+CD8^+$; B cells, $CD19^+$; NK, Natural killer, $CD16^+CD56^+$; timepoint-1 indicates assessment before the third dose of tozinameran. P values relied on parametric two-sided Wald's $\chi 2$ test with Bonferroni (α =0.01) correction for multiple comparisons. A two-sided P value of <0.05 was considered statistically significant.

Supplementary Table 3. Multivariate analysis of peripheral lymphocyte counts at timepoint-2

Covariate	T helper cell count (log)		T cytotoxic cell count (log)		B cell count (log)		NK cell count (log)	
	Beta (95% CI)	P value	Beta (95% CI)	P value	Beta (95% CI)	P value	Beta (95% CI)	P value
Sex								
- male vs. female	0.01 (-0.05 to 0.05)	0.964	-0.05 (-0.12 to 0.01)	0.059	-0.08 (-0.17 to 0.01)	0.052	0.01 (-0.04 to 0.08)	0.577
Age (years)								
- >65 vs. ≤65	0.05 (0.01 to 0.10)	0.012	0.06 (0.01 to 0.11)	0.014	0.03 (-0.03 to 0.10)	0.377	0.09 (-0.03 to 0.14)	0.061
Tumor type								
- breast cancer	-	-	-	-	-	-	-	-
- lung cancer	-0.05 (-0.17 to 0.06)	0.379	-0.04 (-0.17 to 0.09)	0.547	0.03 (-0.15 to 0.21)	0.753	0.01 (-0.12 to 0.15)	0.841
- colorectal cancer	-0.02 (-0.10 to 0.10)	0.973	0.06 (-0.04 to 0.18)	0.245	0.23 (0.07 to 0.39)	0.005	0.04 (-0.07 to 0.16)	0.487
- genitourinary cancer	0.01 (-0.08 to 0.09)	0.963	0.02 (-0.07 to -0.12)	0.589	0.18 (0.04 to 0.32)	0.011	0.06 (-0.03 to 0.17)	0.213
- others	0.02 (-0.04 to 0.09)	0.518	0.02 (-0.05 to 0.10)	0.552	0.09 (-0.02 to 0.20)	0.114	0.05 (-0.03 to 0.13)	0.244
ECOG PS								
- 0	-	-	-	-	-	-	-	-
- 1	-0.03 (-0.07 to 0.01)	0.220	-0.02 (-0.07 to 0.02)	0.374	0.01 (-0.07 to 0.07)	0.922	0.01 (-0.06 to 0.04)	0.791
- 2	-0.01 (-0.11 to 0.09)	0.836	0.03 (-0.08 to -0.14)	0.603	0.06 (-0.09 to 0.23)	0.424	-0.06 (-0.18 to 0.06)	0.319
Smoking habits								
- ever vs. never	0.02 (-0.02 to 0.07)	0.297	0.03 (-0.01 to 0.09)	0.187	0.07 (-0.01 to 0.14)	0.073	0.02 (-0.03 to 0.07)	0.453
BMI								
- ≥30 vs. <30	0.01 (-0.03 vs. 0.07)	0.508	0.05 (-0.01 to 0.11)	0.115	-0.01 (-0.08 to 0.07)	0.922	0.02 (-0.03 to 0.08)	0.430
Disease staging								
- advanced vs. early stage	0.10 (-0.02 to 0.19)	0.017	0.11 (0.01 to 0.21)	0.025	0.10 (-0.03 to 0.24)	0.155	0.06 (-0.03 to 0.17)	0.206
Treatment setting								
- metastatic vs. (neo)adjuvant	-0.15 (-0.24 to -0.07)	0.001	-0.16 (-0.16 to -0.06)	0.001	-0.19 (-0.33 to -0.05)	0.005	-0.09 (-0.19 to 0.01)	0.087
Type of active treatment								
- none (reference subgroup)	-	-	-	-	-	-	-	-
- targeted therapy	0.06 (-0.01 to 0.13)	0.094	0.02 (-0.05 to 0.10)	0.480	0.12 (0.01 to 0.23)	0.030	0.01 (-0.06 to 0.10)	0.691
- cytotoxic chemotherapy	0.01 (-0.04 to 0.08)	0.579	-0.05 (-0.12 to 0.02)	0.168	0.01 (-0.09 to 0.11)	0.847	-0.08 (-0.15 to 0.05)	0.066
- immune checkpoint inhibitors	0.01 (-0.09 to 0.10)	0.931	0.01 (-0.10 to 0.11)	0.950	0.04 (-0.11 to 0.19)	0.589	0.20 (0.09 to 0.32)	<0.001
- hormonal therapy	0.03 (-0.07 to 0.14)	0.548	0.07 (-0.05 to 0.19)	0.268	0.08 (-0.09 to 0.26)	0.360	-0.01 (-0.14 to 0.11)	0.834
- cytotoxic chemotherapy and targeted agents	0.06 (-0.03 to 0.17)	0.187	0.02 (-0.09 to 0.13)	0.695	0.06 (-0.09 to 0.22)	0.454	0.01 (-0.10 to 0.13)	0.808
Corticosteroid therapy [a]								

- yes vs. none	-0.09 (-0.15 to -0.02)	0.005	-0.03 (-0.11 to 0.03)	0.272	-0.12 (-0.22 to -0.02)	0.017	-0.04 (-0.12 to 0.02)	0.222
Khorana score [b]<br>- high vs. low risk	0.01 (-0.07 to 0.09)	0.832	0.01 (-0.08 to 0.10)	0.852	-0.08 (-0.21 to 0.05)	0.223	-0.07 (-0.17 to 0.02)	0.142
Previous TEEs<br>- yes vs. none	0.11 (0.04 to 0.18)	0.001	0.05 (-0.02 to 0.12)	0.202	0.10 (-0.01 to 0.21)	0.064	0.16 (-0.05 to 0.14)	0.071
CVC<br>- yes vs. no	0.03 (-0.01 to 0.08)	0.190	0.06 (0.01 to 0.11)	0.041	0.04 (-0.04 to 0.12)	0.307	0.03 (-0.03 to 0.09)	0.325
Antiplatelet agents [c]<br>- yes vs. no	0.01 (-0.04 to 0.05)	0.773	-0.03 (-0.08 to 0.02)	0.277	0.01 (-0.07 to 0.08)	0.887	0.02 (-0.03 to 0.07)	0.509
Anticoagulation therapy [d]<br>- yes vs. no	-0.01 (-0.07 to 0.04)	0.604	0.02 (-0.04 to 0.08)	0.486	0.02 (-0.06 to 0.10)	0.654	-0.01 (-0.07 to 0.05)	0.806

Log, logarithmic values; CI, confidence intervals; ECOG PS, Eastern Cooperative Oncology Group Performance Status; BMI, body mass index; TEEs, thromboembolic events; CVC, central venous catheter. [a] Corticosteroid therapy indicates ≥10 mg daily of prednisone or equivalent for at least 7 days before the third dose of tozinameran (excluding premedication for chemotherapy); [b] adapted from Khorana score (cancer-related high risk for venous thromboembolism included active diagnosis of gastric, esophagus, lung, pancreatic, gynecological, germ-cell, kidney, and bladder tumors; low risk types for venous thromboembolism include other conditions of solid neoplasm diagnosis); [c] antiplatelet therapies included the use of any dose aspirin, ticlopidine, clopidogrel, or ticagrelor; [d] anticoagulation therapies included the use of any dose low-molecular-weight heparin, apixaban, edoxaban, rivaroxaban, or dabigatran etexilate; T helper cells, $CD3^+CD4^+$ cells; T cytotoxic cell, $CD3^+CD8^+$; B cells, $CD19^+$; NK, Natural killer, $CD16^+CD56^+$; timepoint-2 indicates assessment four weeks after the third dose of tozinameran. P values relied on parametric two-sided Wald's $\chi 2$ test with Bonferroni (α =0.01) correction for multiple comparisons. A two-sided P value of <0.05 was considered statistically significant.

Supplementary Figure S1. Patients' flow and main outcomes

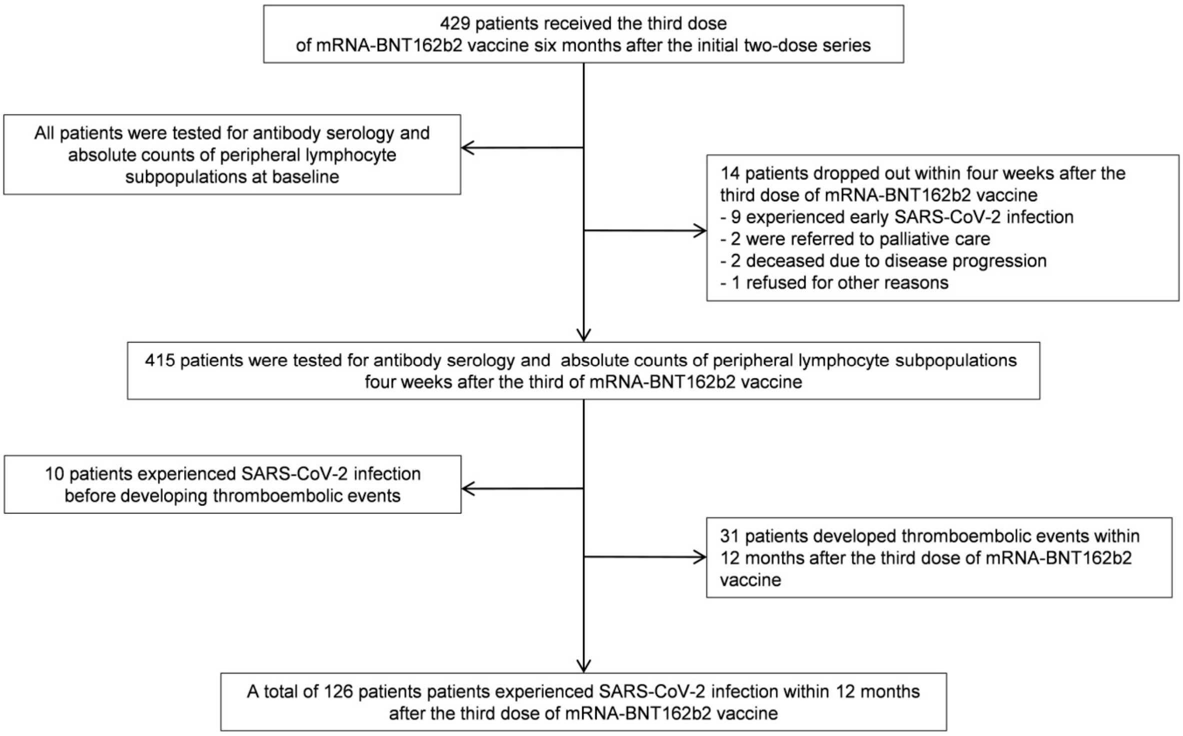

Supplementary Figure S2. Comparison of scatter plot distributions and medians of antibody titers within general population

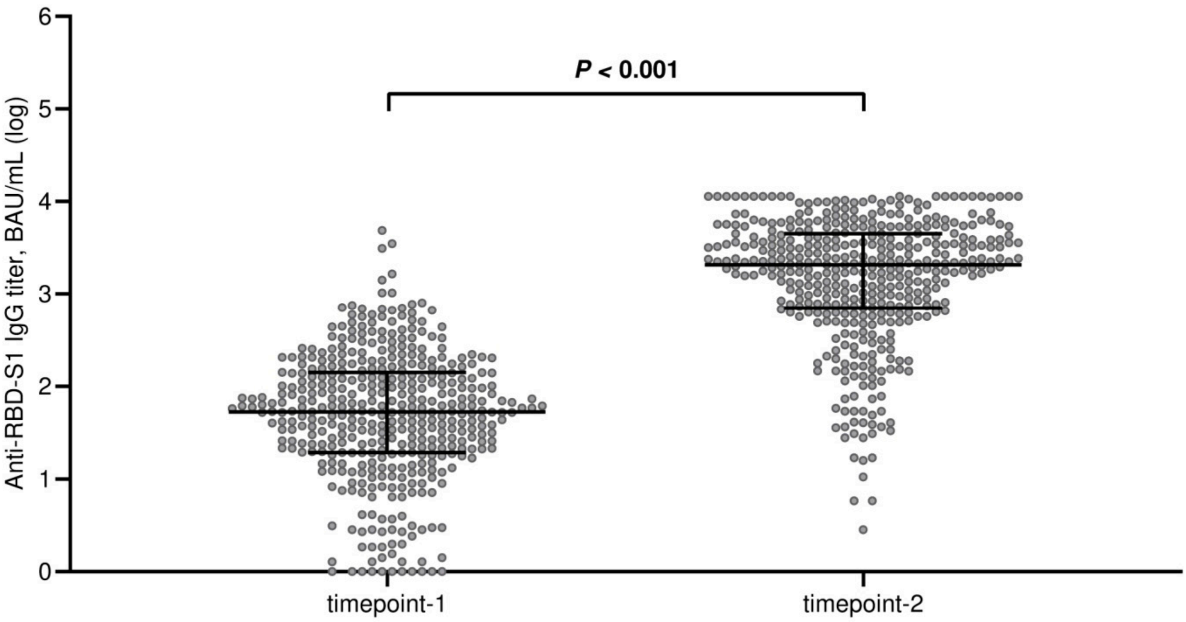

RBD-S1, receptor-binding domain (RBD) of the SARS-CoV-2 Spike protein (S1); BAU, binding arbitrary unit; log, logarithmic value. Bars represent median values with interquartile range; timepoint-1 denotes assessment before the third dose of tozinameran; timepoint-2 denotes assessment four weeks after the third dose of tozinameran.

Supplementary Figure S3. Comparison of scatter plot distributions and medians of antibody titers by treatment types

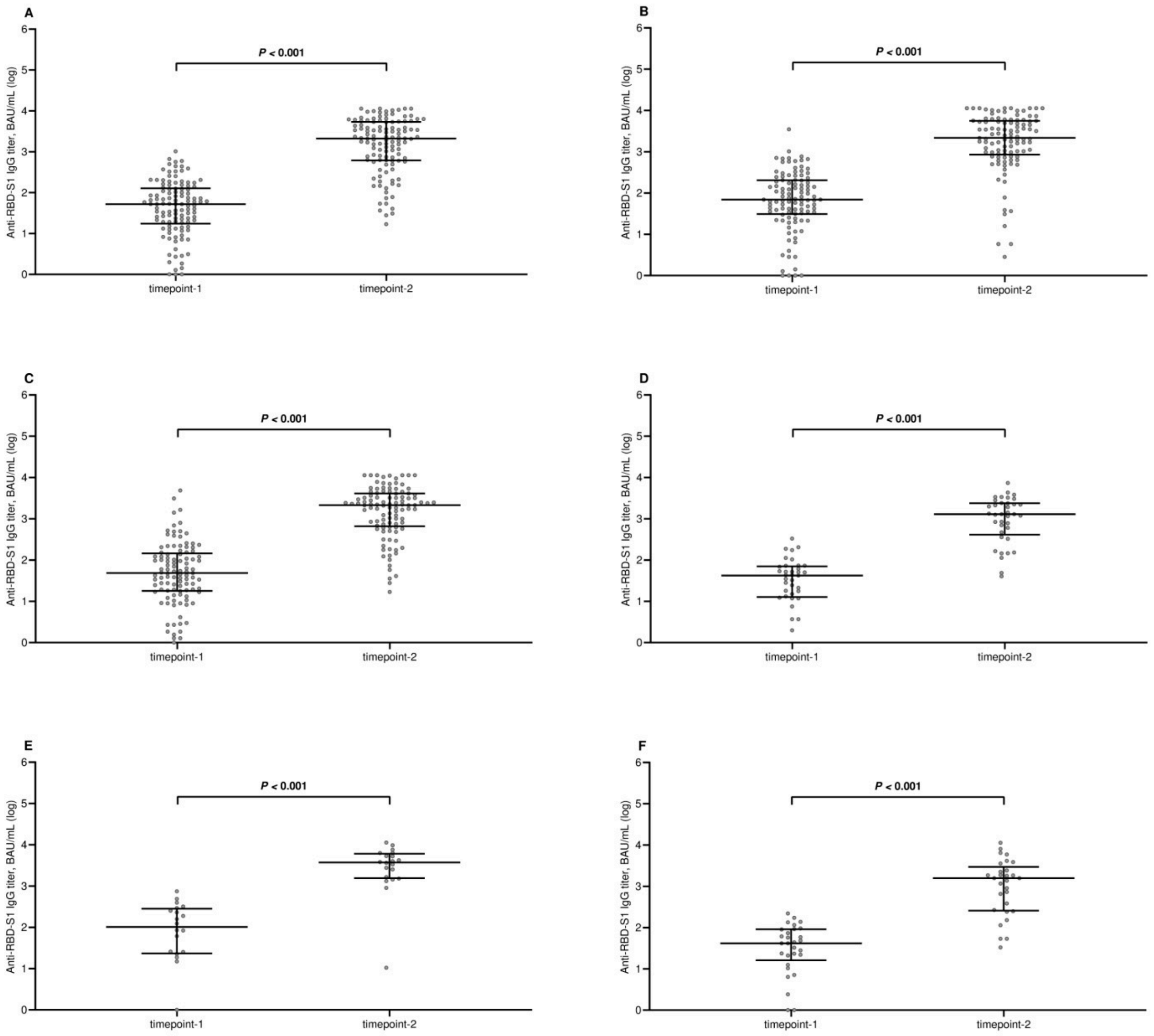

(A) reference subgroup; (B) targeted therapies; (C) cytotoxic chemotherapy; (D) immune checkpoint inhibitors; (E) hormonal therapies; (F) chemotherapy and targeted agents. Log, logarithmic value. Bars represent median values with interquartile range; timepoint-1 denotes assessment before the third dose of tozinameran; timepoint-2 denotes assessment four weeks after the third dose of tozinameran.

Supplementary Figure S4. Changes in peripheral lymphocyte subpopulations within general population

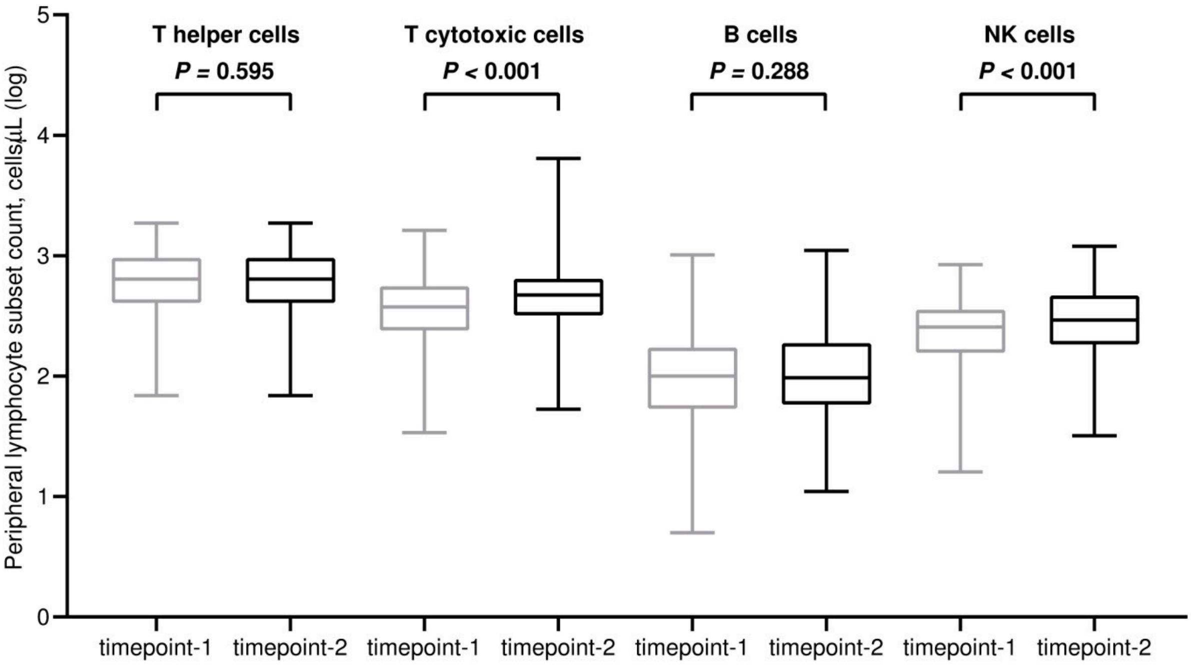

Log, logarithmic value. T helper cells, $CD3^+CD4^+$ cells; T cytotoxic cell, $CD3^+CD8^+$; B cells, $CD19^+$; NK, Natural killer, $CD16^+CD56^+$; bars represent median values with interquartile range; timepoint-1 denotes assessment before the third dose of tozinameran; timepoint-2 denotes assessment four weeks after the third dose of tozinameran.

Supplementary Figure S5. Changes in peripheral lymphocyte subpopulations by treatment types

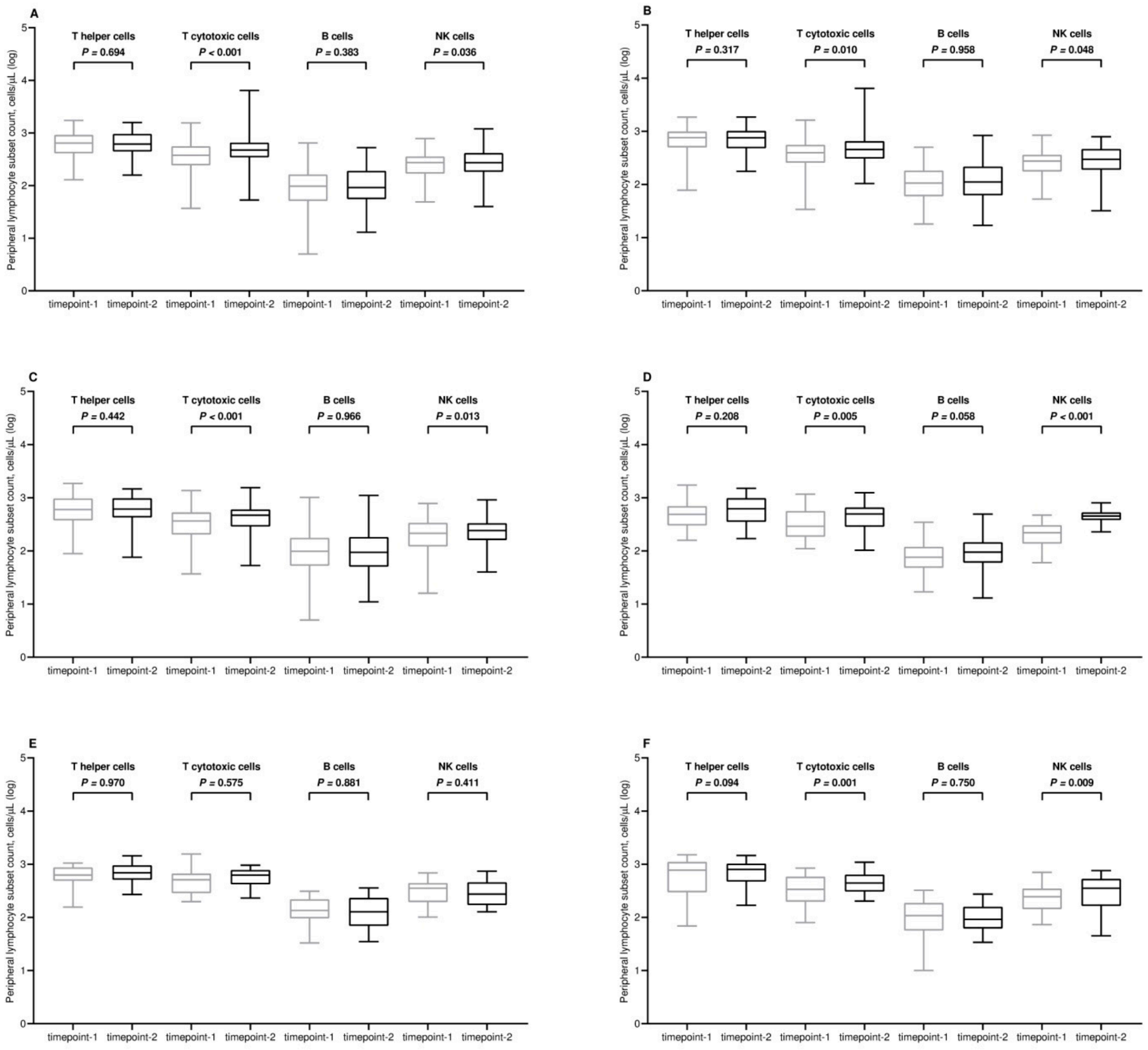

(A) reference subgroup; (B) targeted therapies; (C) cytotoxic chemotherapy; (D) immune checkpoint inhibitors; (E) hormonal therapies; (F) chemotherapy and targeted agents. Log, logarithmic value. T helper cells, $CD3^{+}CD4^{+}$ cells; T cytotoxic cell, $CD3^{+}CD8^{+}$; B cells, $CD19^{+}$; NK, Natural killer, $CD16^{+}CD56^{+}$; bars represent median values with interquartile range; timepoint-1 denotes assessment before the third dose of tozinameran; timepoint-2 denotes assessment four weeks after the third dose of tozinameran.
